# Vitamin D Status at Birth and Future Risk of Attention Deficit/Hyperactivity Disorder (ADHD)

**DOI:** 10.1371/journal.pone.0140164

**Published:** 2015-10-28

**Authors:** Peik Gustafsson, Lars Rylander, Christian H. Lindh, Bo A. G. Jönsson, Amanda Ode, Per Olofsson, Sten A. Ivarsson, Anna Rignell-Hydbom, Nils Haglund, Karin Källén

**Affiliations:** 1 Child and Adolescent Psychiatry, Department of Clinical Sciences, Lund University, Lund, Sweden; 2 Division of Occupational and Environmental Medicine, Lund University, Lund, Sweden; 3 Department of Clinical Sciences, Malmö, Department of Obstetrics and Gynaecology, Skåne University Hospital, Lund University, Malmö, Sweden; 4 Department of Clinical Sciences, Unit of Pediatric Endocrinology, Lund University/CRC, Malmö, Sweden; 5 Department of Clinical Sciences, Lund, Department of Obstetrics and Gynaecology, Reproduction Epidemiology, Lund University, Lund, Sweden; University of Frankfurt, GERMANY

## Abstract

**Objective:**

To investigate whether children with Attention Deficit/Hyperactivity Disorder have lower levels of Vitamin D_3_ at birth than matched controls.

**Material:**

Umbilical cord blood samples collected at birth from 202 children later diagnosed with Attention Deficit/Hyperactivity Disorder were analysed for vitamin D content and compared with 202 matched controls. 25-OH vitamin D_3_ was analysed by liquid chromatography tandem mass spectrometry.

**Results:**

No differences in cord blood vitamin D concentration were found between children with Attention Deficit/Hyperactivity Disorder (median 13.0 ng/ml) and controls (median 13.5 ng/ml) (p = 0.43). In a logistic regression analysis, Attention Deficit/Hyperactivity Disorder showed a significant association with maternal age (odds ratio: 0.96, 95% confidence interval: 0.92–0.99) but not with vitamin D levels (odds ratio: 0.99, 95% confidence interval: 0.97–1.02).

**Conclusion:**

We found no difference in intrauterine vitamin D levels between children later developing Attention Deficit/Hyperactivity Disorder and matched control children. However, the statistical power of the study was too weak to detect an eventual small to medium size association between vitamin D levels and Attention Deficit/Hyperactivity Disorder.

## Introduction

Vitamin D deficiency is a common condition worldwide [1,2]. Although exposure of the skin to sunlight leads to vitamin D production from cholesterol, vitamin D deficiency is common, also in areas rich in sunshine [1,2]. Several hypotheses concerning the importance of vitamin D for brain development, especially prenatally and during the early neonatal period, have been formulated. [3,4]. It has been proposed that vitamin D deficiency could be a risk factor for developing attention deficit/hyperactivity disorder (ADHD) [5,6]. ADHD affects 2–9% of all children and adolescents [7,8], and is a multifactorial condition with high heritability and with underlying environmental risk factors [7,9]. Results from two recent studies on the association between vitamin D status and ADHD diagnosis showed an association between low levels of D vitamin and an ADHD diagnosis [5,6]. In these studies D vitamin levels were not assessed at pregnancy or birth but in children 5–18 years of age. Three studies measuring D vitamin levels in the mothers at pregnancy have not found any association between low levels of D vitamin in pregnancy and that ADHD in the offspring [10–12]. These studies have assessed D vitamin levels in the mothers at pregnancy, but not the actual D vitamin levels of the child at birth. Even if it is probable that the vitamin D levels of the mother is close to the levels of the foetus, it is of interest to compare these results with a study of vitamin D levels in the child as a hypothetical risk factor for later development of ADHD. The levels of vitamin D at birth should be a good estimate of the child’s vitamin D levels, at least in the later part of the gestation.

### Aim

The aim of the study was to test the hypothesis that low levels of vitamin D in the child during pregnancy are a risk factor for ADHD in the offspring.

## Material and Methods

### Children with an ADHD diagnosis

The selection procedure of children diagnosed with ADHD has previously been described [13]. Briefly, 419 children born in the city of Malmö between 1978 and 2000 were diagnosed with ADHD at the Department of Child and Adolescent Psychiatry in Malmö. The diagnosis was made by one of ten experienced clinicians at the department using the Diagnostic and Statistical Manual of Mental Disorders (DSM). The child’s intelligence and ability to concentrate was tested by the Wechsler Intelligence Scale (WISC), the TEA-Ch, and sometimes the QB-Tech or IVA+. A clinical examination, including assessment of neurological soft–signs, was also performed. The teacher and the parents were asked to fill in questionnaires: the SNAP-IV or the Conner’s questionnaire, the 5–15 questionnaire, and the BRIEF -questionnaire. The child’s behaviour at school and at the visits to the clinic was observed and recorded. A team comprising a doctor, a psychologist and sometimes a social worker arrived at a consensus decision concerning the diagnosis on the bases of DSM criteria. The DSM criteria in the DSM-III-R were used before 1994, and the DSM-IV was used after1994. Age at the time of diagnosis varied between 5 and 17 years, with most children being diagnosed between 8 and 12 years. After exclusion of the children who were either not born in Malmö, not linked to the Swedish Medical Birth Registry (SMBR) or for whom no umbilical blood serum was stored, 202 children remained. Descriptive data for the study population are shown in Table 1.

**Table 1:** Descriptive data for the study population. Children with ADHD and controls were matched for age and country of birth of the mother.

	ADHD		Controls		Total	
	n	(%)	n	(%)	n	(%)
Gender:						
Males	179	(88.6)	161	(79.7)	340	(84.2)
Females	23	(11.4)	41	(20.3)	64	(15.8)
Time period:						
1978–1979	1	(0.5)	1	(0.5)	2	(0.5)
1980–1984	9	(4.5)	5	(2.5)	14	(3.5)
1985–1989	68	(33.7)	72	(35.6)	140	(34.7)
1990–1994	98	(48.5)	96	(47.5)	194	(48.0)
1995–1999	26	(12.9)	28	(13.9)	54	(13.4)
Gestational age (weeks):						
<32	5	(2.5)	1	(0.5)	6	(1.5)
32–36	6	(3.0)	6	(3.0)	12	(3.0)
37–41	148	(73.3)	135	(66.8)	283	(70.0)
42+	43	(21.3)	60	(29.7)	103	(25.5)
Birth weight (g):						
<1500	4	(2.0)	0	(0.0)	4	(1.0)
1500–2499	9	(4.5)	5	(2.5)	14	(3.5)
2500–4499	184	(91.1)	193	(95.5)	377	(93.3)
4500+	5	(2.5)	4	(2.0)	9	(2.2)
Maternal age (years):						
<20	8	(4.0)	5	(2.5)	13	(3.2)
20–24	61	(30.2)	42	(20.8)	103	(25.5)
25–29	71	(35.1)	74	(36.6)	145	(35.9)
30–34	39	(19.3)	53	(26.2)	92	(22.8)
35–39	17	(8.4)	19	(9.4)	36	(8.9)
> = 40	6	(3.0)	9	(4.5)	15	(3.7)
Maternal country of birth:						
Sweden	168	(83.2)	169	(83.7)	337	(83.4)
Other	34	(16.8)	33	(16.3)	67	(16.6)
Maternal smoking:						
Unknown	6	(4.0)	8	(4.0)	16	(4.0)
No	123	(63.4)	133	(68.6)	256	(66.0)
1–9 cig/day	34	(17.5)	35	(18.0)	69	(17.8)
>9 cig/day	37	(19.1)	26	(13.4)	63	(16.2)
Maternal BMI:						
Unknown	141	(69.8)	141	(69.8)	282	(69.8)
<18.5	1	(1.6)	3	(4.9)	4	(3.3)
18.5–24.9	36	(59.0)	40	(65.6)	76	(62.3)
25.0–29.9	16	(26.2)	14	(23.0)	30	(24.6)
> = 30.0	8	(13.1)	4	(6.6)	12	(9.8)
Season of birth						
Unknown	0	(0.0)	0	(0.0)	0	(0.0)
January-March	37	(18.3)	32	(15.8)	69	(17.1)
April-June	40	(19.8)	49	(24.3)	89	(22.0)
July-September	53	(26.2)	37	(18.3)	90	(22.3)
October-December	72	(35.6)	84	(41.6)	156	(38.6)

### Serum samples

Using the personal identification numbers, umbilical cord serum samples for children with ADHD were retrieved from the Malmö Maternity Unit Biobank (MMUB). Almost all child deliveries in Malmö take place at the Skåne University Hospital Maternity Unit, where maternal and umbilical cord blood samples have been collected and stored in the MMUB. Maternal blood was collected by venepuncture, a few hours prior to delivery, and cord blood was collected by drainage into sterile sample tubes immediately after birth. The sample tubes were stored overnight in a refrigerator at +8°C for sedimentation. The following morning, the serum was collected and frozen in polypropylene plastic test tubes at -20°C until analysis.

### Control children

A pool of 10 eligible controls per child with ADHD were retrieved from the Swedish Medical Birth Register (SMBR) [14] and matched for year of birth (±12 months) and country of birth of the mother. Certain case-control pairs could thus be close to each other in age, but be borne in different years. The first newborn in the pool of controls with an available umbilical blood sample in the MMUB was chosen. The sample of the next baby born was chosen if none of the 10 control newborns had an available umbilical serum sample in the biobank. Maternal sera for the controls were retrieved as well. Demographic and obstetric information on the mothers and the infants was obtained from the SMBR by using their personal identification numbers.

### Analysis of vitamin D concentration

Quantitative analyses of cord sera were conducted using a triple quadrupole linear ion trap mass spectrometer (QTRAP 5500; AB Sciex, Foster City, CA, USA) coupled to a liquid chromatography system (UFLCXR, Shimadzu Corporation, Kyoto, Japan; LC/MS/MS). Vitamin D concentrations were determined by a modified method for analysis of perfluorinated compounds [15]. Aliquots of 60 μl of serum were digested using glucuronidase and the proteins were precipitated using acetonitrile. The samples were prepared and analysed in 96-well plates. The calibration standard 25-hydroxyvitamin D_3_ and the internal standard D_6_-25-hydroxyvitamin D_3_ were obtained from Toronto Research Chemicals, Inc. (North York, Canada). In all analytical batches there were two different quality control (QC) samples prepared in- house and chemical blanks analysed. The control samples were checked against controls from Chromsystems Instruments & Chemicals GmbH (MassCheck®; Gräfelfing, Germany) and the DEQAS Vitamin D External Quality Assessment Scheme (Endocrine Laboratory, Charing Cross Hospital, UK). The samples were prepared and analysed in random order in triplicates and the mean value of the two closest concentrations was used in the statistical analyses. The variation coefficient for these analyses was 13%.

### Ethics Statement

The study protocol followed the requirements of the Declaration of Helsinki and was approved by the Research Ethics Committee at Lund University, Sweden (Reg.no. 2011/724)

### Statistical analyses

The Wilcoxon matched-pairs signed-rank test was used to compare the vitamin D concentrations between children with ADHD and matched control children. The Kruskal-Wallis/Wilcoxon test was used to compare vitamin D levels stratified for possible confounders. ANOVA analyses were performed concerning differences in the means for vitamin D in children with ADHD compared to control children, stratified for all cases and for children with a mother born in Sweden. A possible linear relationship between vitamin D levels and the risk of ADHD was investigated using logistic regression analysis. The odds ratio (OR) with 95% confidence interval (CI) was calculated. A spline analysis was performed which did not detect any possible threshold for the vitamin D–ADHD analyses. The 10^th^ and 25^th^ percentiles of vitamin D levels among controls were arbitrarily chosen to define possible thresholds. Quartiles of vitamin D levels were also analysed. The possible associations between vitamin D levels below the thresholds and ADHD were tested using conditional logistic regression. Maternal data were analysed as risk factors for ADHD with conditional univariable logistic regression and variables with p values less than 0.20 were considered as potential confounders in a final multivariable conditional logistic regression analysis. The covariates analysed were maternal age, maternal smoking, season of birth, maternal BMI, parity and maternal height, see Table 1 for descriptive data.

#### Power considerations

With 202 children with ADHD and controls matched 1:1, and α = 0.05, the study had an 80% chance of detecting a difference of 0.3 SD. The lowest detectable OR for a value below the 25^th^ percentile was 1.8.

## Results

**Fig 1:**
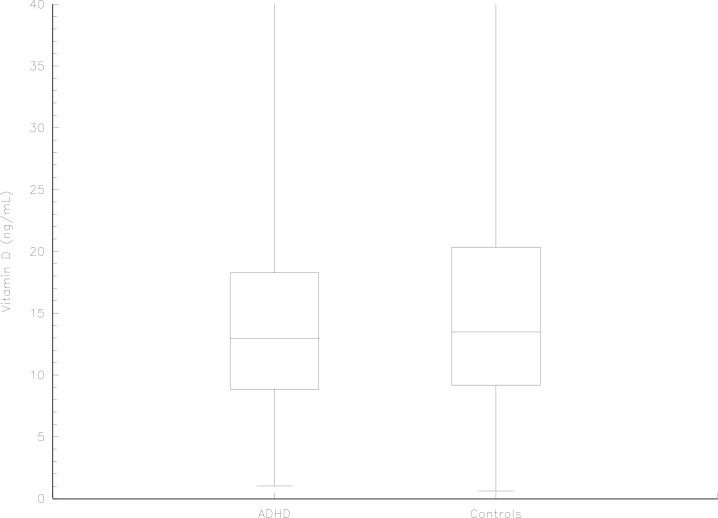
Vitamin D concentrations in individuals with ADHD and controls. Box plot of vitamin D concentrations in serum at birth for individuals with ADHD compared with matched controls. The vitamin D concentrations are measured in ng/ml. The box plot shows medians and quartiles. The difference is non-significant when tested with Wilcoxon’s signed rank test.

The matching of the time of birth showed no heterogeneity between birth periods between cases and controls (p = 0.85, Chi2(4df)), indicating only small differences in the storage time of the samples from cases and controls. Analyses stratified for sex, season of birth, maternal smoking and maternal BMI were performed, see Table 2. Analyses concerning the country of birth of the mother were also performed, see Table 3. No difference between vitamin D levels was detected when the ADHD group (n = 202) was compared with the control group (n = 202, p = 0.43) (Fig 1). A logistic regression analysis revealed no linear association between vitamin D levels and ADHD. The OR for an increase by one unit (ng/ml) in the vitamin D concentration was 0.99 (95% CI: 0.96–1.01). The 25^th^ vitamin D concentration percentile among controls was 9.17 ng/ml. No association between a value below this threshold and ADHD could be detected (OR: 1.13; 95% CI: 0.69–1.83); nor could an association between a vitamin D value below the 10^th^ percentile (5.84 ng/ml) and ADHD be detected (OR: 1.14; 95% CI: 0.56–2.34). The association between vitamin D values in the lower quartile among the Swedish controls (<10.4 ng/ml) and ADHD was also analysed, but was not statistically significant (OR: 1.10; 95% CI: 0.71–1.71). Two seasonal periods had p-values lower than 0.20. When different maternal data (age, BMI, parity, smoking, height) were analysed as risk factors for ADHD, only smoking and maternal age had p- values less than 0.20. In the conditional multivariable regression analysis, only maternal age and smoking had a p- value less than 0.20, so the final conditional logistic regression model was performed with maternal age, maternal smoking, two seasonal periods of birth and vitamin D levels as independent variables. Maternal age (OR: 0.95; 95% CI: 0.91–0.99) and season of birth July to September (OR: 2.30; 95% CI: 1.27–4.16) showed a significant association with ADHD (OR: 0.96; 95% CI: 0.92–0.99), but not smoking (OR: 1.17; 95% CI: 0.89–1.53), season of birth January to March (OR: 1.45; 95% CI: 0.79–2.65) or vitamin D levels (OR: 0.98; 95% CI: 0.95–1.01).

**Table 2:** Descriptive data concerning vitamin D levels (ng/ml) among different subgroups.

	All children		Controls		Children with ADHD	
	n	Mean (SD)	n	Mean (SD)	n	Mean (SD)
Gender:						
Boys	340	14.77 (7.95)	161	15.40 (8.27)	179	14.21 (7.64)
Girls	64	14.08 (7.55)	41	13.68 (7.78)	23	14.80 (7.23)
Season of birth:						
January-March	69	10.41 (6.02)	32	11.17 (6.68)	37	9.74 (5.40)
April-June	89	14.55 (7.29)	49	14.01 (6.90)	40	15.22 (7.79)
July-September	90	20.81 (9.07)	37	22.55 (10.10)	53	19.60 (8.15)
September-December	156	13.06 (5.98)	84	13.83 (6.37)	72	12.16 (5.39)
Maternal smoking:						
No	256	15.52 (8.56)	133	16.20 (8.91)	123	14.79 (8.12)
Yes	132	13.07 (6.35)	61	12.64 (6.02)	71	13.44 (6.63)
Maternal age (years):						
<20	10	12.30 (5.31)	4	10.36 (2.58)	6	13.6 (6.46)
20–24	96	12.46 (6.80)	38	12.47 (6.93)	58	12.46 (6.77)
25–29	141	15.83 (8.39)	72	17.03 (9.07)	69	14.56 (7.47)
30–34	91	15.77 (8.81)	52	16.07 (8.65)	39	15.38 (9.13)
35–39	35	14.71 (6.50)	19	13.46 (5.39)	16	16.19 (7.53)
> = 40	15	13.21 (7.03)	9	10.32 (5.97)	6	17.56 (6.60)
Maternal BMI:						
<18.5	4	11.24 (7.01)	3	9.70 (7.72)	1	15.86(0.0)
18.5–24.9	75	15.94 (7.48)	39	15.89 (7.30)	36	16.01 (7.77)
25.0–29.9	30	14.66 (7.98)	14	17.68 (8.11)	16	12.02 (7.08)
> = 30	12	13.04 (7.26)	4	15.26 (8.82)	8	11.93 (6.73)

**Table 3:** Difference in vitamin D levels (ng/ml) for children with ADHD and control children, stratified for the mother’s country of birth. Analysis with ANOVA according to four models. Model 1: crude unpaired, model 2: crude paired, model 3: adjusted unpaired and model 4: adjusted paired. Adjustment was made for the covariates age of mother, smoking of mother, season of birth January to March and season of birth July to September.

	All Children			Children with mothers born in Sweden		
	estimate	95% confidence limits	p-value	estimate	95% confidence limits	p-value
model 1	-0.77	-2.32, 0.77	0.32	-1.11	-2.76, 0.53	0.18
model 2	-0.77	-2.25, 0.70	0.30	-1.08	-2.77, 0.62	0.21
model 3	-1.00	-2.40, 0.39	0.16	-1.00	-2.47, 0.46	0.18
model 4	-0.99	-2.34, 0.36	0.15	-1.11	-2.66, 0.44	0.16

## Discussion

Our study fails to support the hypothesis that low levels of vitamin D during pregnancy are a risk factor for ADHD in the offspring. No other study of vitamin D levels in children, with a prospective or pseudo-prospective design, has previously been performed. In a study from Qatar, vitamin D in serum was determined in 1331 ADHD children age 5–18 years and in the same number of controls. The ADHD children had lower levels than the controls [5]. In a study from Turkey, 60 ADHD children 7–18 years of age were compared with 30 children without a diagnosis. That study also showed that children with ADHD had lower levels of vitamin D in serum [6]. These results are contradicted by Tolppanen et al. [16] who, in a large English study, found no association between low levels of vitamin D and behavioural problems, including inattention and hyperactivity. Mc Cann et al. [17] reviewed the animal research literature and concluded that there are indications of behavioural effects of vitamin D inadequacy, but that the evidence is weak. Human studies have indicated that vitamin D deficiency is associated with an increased risk of developing many diseases and disorders, like Parkinson’s disease, epilepsy, depression, multiple sclerosis, schizophrenia, autism, and autoimmune diseases like rheumatoid arthritis and type I diabetes mellitus [18–20]. From a theoretical point of view, in foetal life and during early childhood, the immature brain should be more vulnerable to environmental influences like vitamin D deficiency [3,4]. The studies by Gale et al. [10] and by Whitehouse et al. [11] showed that the mother’s vitamin D status during pregnancy had no association with behavioural problems in the offspring, including symptoms of ADHD, several years later. Strøm et al [12] made a follow-up study of children up to 22 years after birth and did not find an association between low D vitamin levels in the serum of the mothers at week 30 of the pregnancy and ADHD in the offspring. While Strøm et al have analysed the levels of D vitamin in the serum from the mothers at pregnancy, we have analysed D vitamin levels in the serum collected from the child at birth, which should reflect the prenatal vitamin D status of the child. We have then compared children who later received an ADHD diagnosis with controls. This means that our study, like the studies by Strøm et al., Gale et al., and Whitehouse et al [10–12] but unlike the recently published studies in Quatar and Turkey [5,6], has a prospective design where vitamin D levels are measured at a time before the child receives a diagnosis. ADHD has a heritability of about 80% [7], but genetically mild forms of ADHD may be sensitive to environmental factors augmenting the symptoms. Vitamin D deficiency acquired at a later age could possibly enhance ADHD symptoms, so that an otherwise mild form of ADHD develops into overt symptoms. Other hypothetical explanations of the findings in the studies from Qatar and Turkey are that children with ADHD eat more junk food, take medication that decreases their appetite, spend more time indoors with computers and are often awake late at night. Such lifestyle factors could lead to lower levels of vitamin D.

### Limitations

This is a medium- sized study and a weak association with the risk of developing ADHD with vitamin D deficiency could not be excluded. The children with ADHD were diagnosed at the clinic for child and adolescent psychiatry, which means that the diagnoses are clinical and do not follow a research study protocol. The diagnoses were made more than ten years ago, and it is probable that the children diagnosed at that timed had more marked symptoms of ADHD than many have today, as the diagnostic criteria have changed towards a more liberal attitude. We have only analysed one single blood sample taken from the umbilical cord at birth from each child. This may not reflect vitamin D levels over time. In a study from 2013 by Major et al. of 583 adult individuals [21], single blood samples from the same individual obtained in the spring and autumn were compared and it was found that a single blood sample provided a reasonable average for 25(OH) D vitamin over a one year period. They concluded that additional studies in younger individuals are needed.

### Conclusion

We found no support for the hypothesis that low levels of vitamin D during pregnancy are a risk factor for ADHD in the offspring. As a consequence of the limited statistical power, we cannot exclude that the may be a small to medium sized association between vitamin D levels at birth and a later diagnosis of ADHD.
